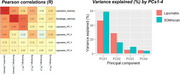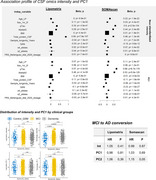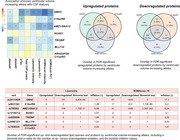# Integrative multi‐omics analysis reveals physiological and genetic drivers of CSF biomarker variability: implications in neurodegeneration studies

**DOI:** 10.1002/alz70856_099623

**Published:** 2025-12-25

**Authors:** Pablo García‐González, Raquel Puerta, Jonas Dehairs, Itziar de Rojas, Laura Montrreal, Marta Marquié, Maitee Rosende‐Roca, Asif Emon, María Eugenia Sáez, Bart Smets, Adelina Orellana, Lluís Tárraga, Mercè Boada, Johannes V Swinnen, Victoria Fernández, Alfredo Cabrera Socorro, Agustin Ruiz

**Affiliations:** ^1^ Ace Alzheimer Center Barcelona – International University of Catalunya (UIC), Barcelona, Spain; ^2^ Laboratory of Lipid Metabolism and Cancer, Department of Oncology, KU Leuven, Lueven, Belgium; ^3^ Ace Alzheimer Center Barcelona‐Universitat Internacional de Catalunya, Barcelona, Spain; ^4^ CIBERNED, Network Center for Biomedical Research in Neurodegenerative Diseases, National Institute of Health Carlos III, Madrid, Spain; ^5^ Janssen Research & Development, A Division of Janssen Pharmaceutica, Neuroscience Therapeutic Area, Beerse, Belgium; ^6^ CAEBI. Centro Andaluz de Estudios Bioinformáticos, Sevilla, Spain; ^7^ Ace Alzheimer Center Barcelona – International University of Catalunya (UIC), Barcelona, Barcelona, Spain; ^8^ Ace Alzheimer Center Barcelona‐Universitat Internacional de Catalunya, Barcelona, Barcelona, Spain; ^9^ Glenn Biggs Institute for Alzheimer's & Neurodegenerative Diseases, University of Texas Health Science Center, San Antonio, TX, USA

## Abstract

**Background:**

Cerebrospinal fluid (CSF) biomarkers are key sources of insight for research and clinical practice in the neurodegeneration field. Here, we used omics data to characterize a physiological source of variability which has a major impact in the concentration of CSF analytes and is rarely accounted for in these studies.

**Methods:**

We studied 1,372 samples from the ACE Alzheimer Center Barcelona memory clinic, including cognitively unimpaired subjects and patients with mild cognitive impairment or dementia. We analysed CSF lipidomics (Lipometrix, 386 species), proteomics (SomaScan 7K, 2395 species), and genomics data (TOPMed‐imputed Affymetrix Axiom 815K Array). We estimated the overall concentration (mean standardized values) and principal components (PCs) of the proteomics and lipidomics data independently. Genome‐wide associations (GWAs) were performed using PLINK v2.00a3 adjusting by age, sex and population microstructure. Enrichment analysis was conducted using WebGestalt.

**Results:**

Mean standardized intensities and the PC1 (explaining 40% and 60% of the CSF lipidomics and proteomics variance, respectively) were highly intercorrelated (Figure 1). Despite independent from disease groups and progression, these metrics were strongly associated with CSF *p*‐tau181 and Aβ42 levels (Figure 2). GWAs revealed that *GMNC*, previously associated with CSF *p*‐tau levels and lateral ventricular volume, and linked to multicilliated cell differentiation in the choroid plexus, was associated (*p* <5·10^‐08^) with these metrics in both experiments. Known alleles increasing ventricular volume were negatively associated with the mean intensity and PC1, downregulating a substantial portion of the proteome (Figure 3). Interestingly, we observed a large overlap in both the down‐ and upregulated proteomic signature of these variants. Enrichment analysis revealed that proteins diluted by ventricular volume‐increasing alleles were related to neuronal function, while increased proteins were associated with immune functions, pinpointing CSF production/clearance rates as likely contributors to this phenomenon. These alleles also caused widespread CSF‐specific proteome and metabolome downregulation in an external validation cohort (https://ontime.wustl.edu/).

**Conclusions:**

We demonstrate that the main source of variability in the CSF is a non‐disease related trait traceable to genetic loci and closely related to ventricular volume. Accounting for this physiological variability is essential for accurate interpretation of CSF biomarkers, with broad implications for neurodegeneration research and clinical practice.